# Benefits of Blockchain Initiatives for Value-Based Care: Proposed Framework

**DOI:** 10.2196/13595

**Published:** 2019-09-27

**Authors:** Rongen Zhang, Amrita George, Jongwoo Kim, Veneetia Johnson, Balasubramaniam Ramesh

**Affiliations:** 1 Georgia State University Atlanta, GA United States; 2 Marquette University Milwaukee, WI United States; 3 University of Massachusetts Boston Boston, MA United States

**Keywords:** blockchain, balanced scorecard, evaluation, value-based care

## Abstract

**Background:**

The potential of blockchain technology to achieve strategic goals, such as value-based care, is increasingly being recognized by both researchers and practitioners. However, current research and practices lack comprehensive approaches for evaluating the benefits of blockchain applications.

**Objective:**

The goal of this study was to develop a framework for holistically assessing the performance of blockchain initiatives in providing value-based care by extending the existing balanced scorecard (BSC) evaluation framework.

**Methods:**

Based on a review of the literature on value-based health care, blockchain technology, and methods for evaluating initiatives in disruptive technologies, we propose an extended BSC method for holistically evaluating blockchain applications in the provision of value-based health care. The proposed method extends the BSC framework, which has been extensively used to measure both financial and nonfinancial performance of organizations. The usefulness of our proposed framework is further demonstrated via a case study.

**Results:**

We describe the extended BSC framework, which includes five perspectives (both financial and nonfinancial) from which to assess the appropriateness and performance of blockchain initiatives in the health care domain.

**Conclusions:**

The proposed framework moves us toward a holistic evaluation of both the financial and nonfinancial benefits of blockchain initiatives in the context of value-based care and its provision.

## Introduction

### Background

The health care sector has recently been focused on two related challenges: the transition to value-based care and the use of innovative technologies (such as blockchain technology) to facilitate the delivery of health care. The transition to value-based care, which aims to improve the value of care while providing it at a lower cost, places new demands on health care information systems (IS) [1] that current health information technology infrastructure is not designed to support [1]. Adler-Milstein et al [1] identified three major stakeholder groups that must be supported in achieving value-based care: patients, providers, and researchers. Disruptive technologies such as blockchain offer the potential to support these currently inadequately supported stakeholder groups with Health Information Technology (IT) infrastructure.

Blockchain technology, widely celebrated as a technological revolution, is creating unprecedented hype and optimism [2]. Blockchain is a distributed database that maintains a continuously growing list of data records that are secured from tampering and revision [3,4]. A global survey documents the widespread application of blockchain in domains such as health care, manufacturing, legal, government, not for profit, retail, real estate, tourism, and media [5]. The potential of this technology to aid organizations in achieving strategic goals like value-based care is increasingly being recognized by health care providers and other stakeholders (eg, payers, shareholders, accreditation agencies) [6]. However, Iansiti and Lakhani [7] note that practitioners are uncertain about the impact that disruptive technologies such as blockchain might have on organizational performance. Current research and practice lack comprehensive approaches to evaluating the benefits of blockchain and developing appropriate use cases of blockchain applications for value-based care [8].

As IT is increasingly becoming a strategic necessity for improving services and reducing medical errors [9], comprehensive approaches to evaluating the appropriateness and value of disruptive technologies such as blockchain are needed. An evaluation approach should facilitate the assessment of both technical and nontechnical (eg, legal, data ownership and privacy, security) implications. To address this need, we assessed two sets of existing evaluation frameworks: technology evaluation methods (the Zachman framework, human-computer interaction [HCI] guidelines, and the technology-centric framework) and comprehensive evaluation methods (total quality management [TQM], the European foundation quality management excellence model (EFQMEM), the performance pyramid, and the performance prism). Based on this assessment, we identified deficiencies in the existing evaluation methods and subsequently developed an approach that extends the balanced scorecard (BSC) framework that addresses these deficiencies.

The BSC, developed by Robert Kaplan and David Norton nearly two decades ago [10], provides organizations with a structured approach to assessing both the financial and nonfinancial dimensions of organizational initiatives and processes in terms of strategic outcomes. Beyond the purely accounting-based measures traditionally used, the BSC is balanced in that it provides a comprehensive view of organizational performance. It translates high-level organizational vision and strategy into a holistic set of performance and action measures [11]. The BSC is a practical method that is applicable within the health care service sector and health care organizations, and it has previously been used to assess clinical outcomes, for example [12]. However, it has not yet been used to evaluate disruptive innovations, such as blockchain, that can improve patient care and reduce costs but that have regulatory, financial, and operational implications.

A myriad of seemingly promising blockchain projects are being implemented in the health care domain, often without careful consideration of the applicability of the technology [13]. Moreover, questions still linger for early adopters of this technology: “How does an organization holistically assess the performance of blockchain technology in the health care domain?” and “Does the introduction of blockchain technology align with the strategic priorities of a health care organization?”. Answering these questions is critical for health care organizations to achieve the health care IT mission identified by the US federal government, namely: "Improve the health and well-being of individuals and communities through the use of technology and health information that is accessible when and where it matters most" [14].

We sought to answer the above questions through our assessment of existing evaluation frameworks and the development of a new framework that can guide the comprehensive evaluation of the value of blockchain initiatives that seek to enable the delivery of value-based care.

In the sections below, we first discuss the relevant literature on value-based health care and blockchain technology. We then assess existing evaluation frameworks and present our framework, which extends the BSC by addressing some of its limitations. Further, we customize the framework to the context of blockchain applications in health care settings. We then present an illustrative case study on the application of the framework in a pharmaceutical supply chain organization. Finally, we discuss the implications of our framework for both researchers and practitioners.

### Information Technology Support for Transitioning to Value-Based Health Care

Health care value, defined as health outcomes (including quality of care achieved per dollar spent), has become a cornerstone of the strategy to restructure the US health care system [15-17]. One of the proposed frameworks for improving health care value is the value-based care model [18]. Value-based care attempts to advance the triple aim of providing better care for individuals, improving population health management strategies, and reducing health care costs. Value-based care models center on patient outcomes and how well health care providers can improve quality of care using measures such as reduced hospital readmissions, improved timeliness and safety of care, more equitable care, shared decision-making, and improved preventative care [17]. This model ties payments for care delivery to the quality of care provided, and rewards providers for both efficiency and effectiveness [19].

Unlike more traditional approaches, value-based care is driven by data because providers must report to payers on specific metrics and demonstrate improvement. Providers are required to use IT systems to track and report metrics such as hospital readmissions, adverse events, population health, and patient engagement. Further, providers are incentivized to use evidence-based medicine, engage patients, upgrade health care IT, use data analytics, and receive payments electronically. When patients receive more coordinated, appropriate, and effective care, providers are rewarded. To achieve these goals, health care organizations need a digital infrastructure that facilitates the provision of comprehensive, affordable, accessible, effective, and error-free care.

While significant progress has been made in digitizing the US health care system, today’s health IT infrastructure largely remains a collection of systems that were not designed to support the transition to value-based care [1]. In fact, prior literature has identified a health IT chasm, which refers to the gaps between the current health IT ecosystem (see Multimedia Appendix 1) and the system that is needed for value-based care [1,20-42].

In fact, a recent study identified several gaps from the perspectives of three stakeholder groups. From the patient perspective, patients are unable to access electronic medical records from most providers, and most care providers do not provide functionalities for patients to submit patient-generated data. Only a small percentage of patients receive clinical trial information from their primary physician, and an even smaller percentage participate in biobanks [1]. From the provider perspective, due to the lack of standardized application interfaces providers have difficulty accessing external data, which hinders the advanced analytics on which personalized assistance is based [43]. In addition, manual credentialing (typically takes more than 120 days) and administration of contracts is complicated and inefficient. Further, pharmaceutical providers find it challenging to ensure the authenticity of pharmacy products due to a lack of transparency in current supply chain systems. Finally, from the researchers’ perspective, it is difficult for them to track investigational products to ensure data authenticity, and payments to investigators are delayed due to manual processing [33]. The health IT environment is immature, provides few safeguards for safety and effectiveness, and provides very limited integration of applications used in clinical care or research.

Prior literature has also identified specific goals (eg, improving patients’ access to clinical data, improving patient’s ability to submit and access data via mobile health technology, more readily engaging patients in clinical research) for addressing the needs of each of these stakeholder groups [1]. Blockchain technology may help achieve these goals.

### Blockchain for Enabling Value-Based Care

Blockchain consists of blocks that hold batches of individual transactions. Each block contains a timestamp and a link to a previous block [3,4]. The most salient benefit of blockchain is decentralization and the elimination of a trusted centralized third party in distributed applications. Thus, multiple parties can conduct transactions in a distributed environment without the need for a centralized authority, thereby avoiding a single point of both trust and failure. The absence of a centralized processing entity may reduce time and costs. A consensus mechanism is used to reconcile any discrepancies that may arise between participants in a blockchain network.

Iansiti and Lakhani [7] summarized five basic principles underlying blockchain technology: a distributed database, peer-to-peer transmission, transparency with pseudonymity, irreversibility of records, and computational logic. These unique characteristics of blockchain technology enable the development of solutions that reduce uncertainty and ambiguity and enhance security of stored transactional information by providing full transparency and a single truth for all network participants [44]. Although blockchain technology enjoys the benefits of decentralization, it often comes at the cost of scalability. Blockchains are typically incapable of processing large numbers of transactions in a timely manner [1]. The trustless peer-to-peer network infrastructure, which requires information to be propagated to and validated at each node, is the root of this problem. Several solutions (eg, off-chain transactions, sharding, and a provably neutral cloud) have been proposed to address this issue. For example, Leung et al proposed a design that minimizes storage, bootstrapping costs, and bandwidth costs of joining a network by 90% [45]. Such advances are essential for blockchain to realize its disruptive potential [46]. However, effective management of personal health records using blockchain technology still requires improvements such as reduced data size, strengthened personal information protection, and reduced operational costs [47].

Despite its technological infancy, experimental adoption and customization of blockchain technology appears to be fully underway in the health care domain [8]. One of the most impactful health care applications is expected to be the management of electronic health records (EHRs). The decentralization, immutability, traceability, security, and privacy of blockchain make it well suited for the storing, managing, and sharing of patient-centric data among stakeholders [48-50]. Aligning with the requirements of the European General Data Protection Regulation (GDPR), blockchain can be used to build health care platforms that empower patients to control how their data are used and ensure that sensitive personal data are not revealed without the patients’ consent [2,22,51]. Guardtime [2,51], MedRec [23], the Gem Health Network [44], Patientory, and IBM’s Watson [21] are some of the key projects in this ecosystem.

Another salient application domain of blockchain is supply chain management in the pharmaceutical industry. Because of the immutability and traceability of blockchain, any modification of a prescription by any party in the supply chain can be detected, which, in turn, can help address the severe problem of counterfeit medications [2,44,49]. In addition, in biomedical research and education, blockchain could facilitate the elimination of falsification of data or the exclusion of undesirable results from clinical trials [31]. Benchoufi [38] and Nugent et al [37] illustrated the ability to trace patients’ consent and provide data transparency in clinical trials. Moreover, insurance claim processing is a promising area for blockchain applications because of its transparency, decentralization, immutability, and auditability; a few prototype implementations, such as MIStore [52] and Politdok’s initiative partnered with Intel [53], have been reported [44]. Other promising areas include remote patient monitoring [24] and precision medicine [54].

Blockchain technology has the potential to address some of the gaps in the current health IT ecosystem, thereby supporting the three important stakeholder groups involved in value-based care [1]. Multimedia Appendix 1 identifies these gaps and highlights what blockchain can do to address these gaps. Based on a careful study of the needs of the three stakeholder groups, we further outline in the appendix how specific characteristics of blockchain technology may help meet these needs. We also list some proof-of-concept systems that provide some of the desired functionalities.

## Methods

### Overview

While blockchain offers the potential to address issues (eg, interoperability, difficulty in providing optimal personalized care due to lack of comprehensive medical records, and maintaining integrity of records) that are critical for effective value-based care [55], there is limited research comprehensively evaluating the financial and nonfinancial benefits of blockchain solutions in health care [56]. A review of the literature on value-based health care strongly suggests the need for a framework to holistically evaluate the impacts of technologies such as blockchain. Existing evaluation mechanisms (such as the Level of Information System interoperability reference model [56]) have focused on the operational aspects of blockchain. Motivated by the need for a framework to guide the strategic evaluation of blockchain applications within a health care organization, we extend the BSC approach, which is an already well-established performance evaluation system. Specifically, our approach integrates financial and nonfinancial perspectives (ie, internal processes, learning and growth, external perspectives, and customer perspectives), which are parts of the original BSC, with an external perspective that incorporates the viewpoints of external stakeholders and regulators, especially because of the significant role these parties play in health care delivery. In the following section, we illustrate the use of our framework with a blockchain application for managing a pharmaceutical supply chain.

### Performance Evaluation of Health Care Blockchain Implementations and the Balanced Scorecard

Traditional performance measurement systems have either focused purely on financial factors, ignoring the value of nonfinancial factors [12], or have focused solely on the effectiveness of the technical system without considering the external or financial implications. Health care organizations have been using economic evaluations for health care decision-making for several decades. During this period, increased pressure on health care budgets has necessitated the consideration of cost-effectiveness in addition to clinical effectiveness. Economic evaluation approaches have also been applied to other health care–related decision-making in terms of funding, reimbursement, and new technologies [57,58]. Even comprehensive evaluation approaches that include cost-consequences analysis, cost-minimization analysis, cost-effectiveness analysis, cost-utility analysis, and cost-benefit analysis [59] are focused on financial factors and give limited consideration to nonfinancial aspects of evaluation targets. For example, Zachman’s framework [60] evaluates business-IT alignment in detail but lacks a holistic governance framework. Similarly, the human-computer interaction [61] and technology-centric frameworks [62] provide insights into developing intuitive and interactive IS, but they do not focus on assessing the impact of these systems from external and financial perspectives. Additionally, the interrelationships between the various functional areas in an organization are overlooked in these frameworks. For example, a blockchain implementation in one functional area, such as improving patients’ access to their own medical records, may have major impacts in other areas, such as customer service management, internal processes for quality assurance, security checks, or external partnerships (with, say, insurance companies or pharmacies). Finally, the knowledge that results from the long-term growth of organizations or the ability to deal with future threats also needs to be factored into the performance evaluation [12]. The BSC has dual functions as a performance framework and a management methodology, and thus can tackle the shortcomings of traditional performance measurement systems. These shortcomings include the lack of consideration of nonfinancial factors and the lack of strategic focus. Our evaluation suggests that the BSC addresses both shortcomings and is well suited for the evaluation of disruptive technologies, especially in the dynamic environment in which health care organizations operate.

Our comparison of the various performance measurement systems, as presented in Multimedia Appendix 2, suggests that BSC is an appropriate approach for evaluating blockchain initiatives in achieving value-based care for the following reasons. We compared BSC with two sets of existing methods, namely, technology evaluation methods (the Zachman framework, HCI, and the technology-centric framework) and comprehensive performance evaluation methods (TQM, the European foundation quality management excellence model, the performance pyramid, and the performance prism) [63-65]. Technology evaluation methods typically do not provide a holistic view (such as the consideration of external or customer perspectives) and therefore are not appropriate in our setting. Among the comprehensive evaluation methods, TQM’s narrow focus on internal process is inadequate, and the European foundation quality management excellence model, designed to improve TQM, lacks a strategic focus. Although both the BSC and the performance pyramid use strategic mapping to link strategy to operational metrics, prior research suggests that the performance pyramid is less effective and harder to understand than the BSC [64]. Moreover, although the performance prism considers stakeholders’ perspectives, it does not provide adequate guidelines and neglects to show how the proposed measures can be operationalized [65]. Thus, our comparison of the various technical and comprehensive performance evaluation methods suggests that, among them, the BSC is the most suited to evaluate the performance of disruptive technologies (such as blockchain) in value-based care initiatives.

Organizations in multiple domains, including health care, have adopted the BSC [66,67]. In increasingly dynamic business environments, traditional performance evaluation approaches may not work well due to the uncertainty involved in ascertaining both the costs and benefits of new technologies, such as blockchain. However, both theoretical research and practitioner articles support the use of the BSC for evaluating IT initiatives in such contexts. For example, Gartner [68] notes that performance measurement solutions deployed within an organization should include a spectrum of leading measures rather than focusing on lagging financial indicators. To provide a holistic assessment, Gartner [29] recommends using the BSC to measure return on investment (ROI) and the business value of IT services because it enables the consideration of both financial and nonfinancial perspectives and helps develop relevant metrics [68]. Researchers also recognize the BSC framework as a holistic approach that provides managers with a structure to develop metrics that reflect performance from various perspectives [69], hence our selection of the BSC as the basis for the development of our approach to evaluating blockchain applications.

The BSC measures the performance of organizations from the following four linked and balanced perspectives:

1. Financial: How do we increase value for our shareholders (or providers of financial resources)?
2. Customer: How well do we satisfy our customers’ needs?
3. Internal: How well do we perform key internal operational processes? To satisfy our customers, in what processes must we excel?
4. Learning and growth: Are we able to sustain innovation, change, and continual improvement? Do we have the basic infrastructure in place to improve, create, value, and achieve our mission?

**Figure 1:**
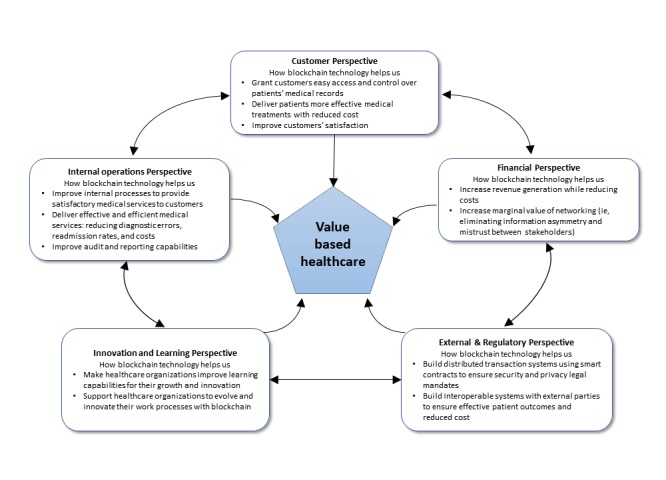
Proposed framework for evaluating blockchain initiatives for value-based care.

Some limitations of the traditional BSC have received attention in the literature [70,71]. One major concern is that the environment external to the organizations, including key groups of stakeholders, is not represented in the framework. For example, Mohobbot [72] points out that the BSC is unable to answer questions concerning the impact of external competitors. Moreover, the BSC does not consider the extended value chain, in which supplier and employee contributions are very significant [73]. This issue is exacerbated in the health care domain due to the complex interactions among the wide variety of organizations and stakeholders that are part of the ecosystem. For example, Norreklit [30] identifies crucial stakeholders like public authorities and suppliers, but other external stakeholders may include insurers, physicians, hospitals, clinics, laboratories, clinical research organizations, supply chain logistics stakeholders (such as pharmaceutical manufacturers, distributors, and retailers), government and regulatory agencies, and charities. To account for the impact of external stakeholders, we extended the BSC with an additional perspective, namely the external and regulatory perspective (see Figure 1). This perspective seeks to answer the following question: "How well does the organization improve value creation through external partnerships while ensuring regulatory compliance?"

By integrating financial measures with other crucial performance indicators concerning patients, organizational learning, growth and innovation, internal processes, and external perspectives, this extended BSC framework offers health care organizations a comprehensive view of the performance of blockchain applications.

## Results

### Summary

In this study we adopted a for-profit health care organization’s view, as a majority of current blockchain implementations are in for-profit organizations.

### Financial Perspective

From a value-based perspective, one of the key questions health care organizations should ask is: "How do health care organizations use blockchain applications to generate more profits at lower cost?" Typically, the focus of the financial perspective in the BSC has been on traditional financial metrics such as ROI and net income. In the context of value-based health care, patient-centric metrics such as gross revenue, adjusted cost per discharge, in-patient or out-patient revenue mix, contract allowances, discounts as a percentage of operating patient revenue [74], patient-payer mix, Medicare or Medicaid mix, average length of stay, and occupancy rate all deserve consideration.

The auditability and traceability features of blockchain enable more secure and efficient revenue management. As it does not require an intermediary, blockchain can support health care financing tasks, such as automatic claims processing using smart contracts [48,75], preauthorization of payments [36], and alternative payment models [76]. A distributed ledger makes claims processing and payment transactions more efficient and cost-effective. Replacing redundant health care intermediaries (namely, organizations that operate between stakeholders and institutions but that add little value to the health care value chain [54]) with transparent blockchain technology could facilitate processes like real-time claims adjudication [75]. With the data provenance benefits offered by blockchain, providers and patients could have enhanced accessibility to patient data. Blockchain technology can also help eliminate information asymmetry and mistrust between stakeholders in the health care ecosystem. With the innate immutability, transparency, and traceability provided by blockchain technology, medical products can be traced from manufacturer to patient, thereby reducing medication and medical equipment fraud. However, in the short-term, the adoption of blockchain technology will likely involve significant investments in application development, and their integration with legacy systems might initially undermine the financial benefit to shareholders.

### Customer (Patient) Perspective

From a value-based care perspective, one of the key questions health care organizations should ask is: "How can we improve our service to customers and satisfy customer needs via blockchain applications?" Improving the performance of health care information systems that support the provision of effective and efficient care to patients is critical for achieving this goal. The patient-centric care paradigm requires the sharing of patients’ EHRs, which raises issues such as privacy, confidentiality, integrity, availability, and security [77]. As a valuable personal asset, health care data should be owned and controlled by customers (patients) easily and securely without violating their privacy [41]. With blockchain-supported applications such as FHIRChain and Blockchain-Based Multi-level Privacy-Preserving Location Sharing Scheme (BMPLS), which simplify data authentication and authorization, patients can control access to their medical data easily and quickly. According to a seminal paper on IS success [78], user satisfaction is affected by information quality, system quality, and service quality. Blockchain enables health care stakeholders to access complete, relevant, and secure data on patients, thereby improving information quality. Health care organizations can overcome common challenges, such as data segregation, and achieve better integration of patients’ medical data. Blockchain supports data immutability and auditability, thereby improving service quality (eg, reliability, responsiveness, and rapport) of medical IS [79], and as a result health care organizations can enhance their medical service quality and thereby patient satisfaction. Blockchain can help health care organizations easily integrate various elements of clinical data, which can enable medical professionals to make accurate diagnoses at low cost.

### Internal Perspective

From a value-based care perspective, one of the key questions that health care organizations should ask is: "What internal processes can blockchain improve to satisfy our customers and the population in general?" Effective internal business processes are critical for providing products and services that satisfy health care organizations’ customers’ needs in a fiscally responsible manner. These effective processes can be reliable indicators of future financial and operational success [12]. With blockchain applications, health care organizations can build time-stamped, tamper-proof, immutable ledger systems that will improve organizations’ auditing and reporting capabilities. These capabilities are crucial for identifying failures in internal processes and remedying those failures. Some benefits that accrue with improvements to internal processes include reduced length of patient stay, accuracy of services provided (both primary and ancillary), optimal surgical capacity utilization, and timeliness of services [12].

A variety of internal processes are candidates for improvements using blockchain technology. Using smart contracts, organizations can encode internal logic (eg, validating identity and tracking the participation of various stakeholders. such as patients and health providers), which will enhance service quality. Service quality can be reflected in measures such as reductions in diagnostic errors, readmission rates, and data security incidents, all of which lower costs. Value for customers can also be improved by instituting newer internal processes, such as Hitech service (eg, digitization of wellness check in Mount Sinai’s Lab 100 [80]). Access to longitudinal medical charts using blockchain applications (such as those implemented in FHIRChain) can help health care organizations achieve optimal results with Hitech services, thereby enabling effective long-term care for chronic illnesses (eg, diabetes). Further, such charts can be useful in designing effective population outreach programs. Finally, using peer-to-peer network-enabled blockchain applications (eg, BMPLS), health care organizations can leverage newer mechanisms of health care delivery, such as telecare, to increase their reach, thereby improving health equity while providing care at a reduced cost.

### External Perspective

From a value-based care perspective, one of the key questions health care organizations should ask is: "How can we leverage external partnerships to create value while ensuring regulatory compliance, thus satisfying our customers and the general population?" Creating effective partnerships with external stakeholders (eg, payers, accreditation bodies) while remaining compliant with regulations is critical for value creation. These partnerships enable health care organizations to supply products and services that satisfy customer needs in a fiscally and legally responsible manner.

Some multi-level, privacy-preserving. location-sharing blockchain applications (eg, BMPLS) enable interoperability with external systems, thereby enabling access to multi-dimensional medical charts from various stakeholders that can improve long-term medical care at a low cost. Through external partnerships, these health care organizations can seek to create value by taking a proactive role in providing care to their customers (say, by tracking customers’ lifestyle and suggesting changes). Naturally, such partnerships can enable future financial and operational success through service innovation, which can help build deeper long-term relationships with customers. Additionally, having access to multi-dimensional population health data will enable health care providers to design outreach services that benefit the community as a whole. Blockchain solutions may also include smart contracts that help meet security and privacy mandates. Further, through the standardization of smart contracts at both the provider’s and the external partner’s end, interoperability of medical systems for value creation can be achieved.

### Learning and Innovation Perspective

From a value-based care perspective, one of the key questions health care organizations should ask is: "How can we use blockchain applications to improve the learning capabilities that lead to growth and innovation?" Blockchain applications can help health care organizations reassess their resources, from employee capabilities to health care delivery processes, and align them to the organization’s strategy.

Blockchain enables health care stakeholders to learn and to improve their services, thereby enhancing their competitiveness and sustainability. The systems interoperability enabled by blockchain technology can help health care professionals learn about opportunities to innovate their services. Blockchain technology also supports organizations in reassessing existing processes and resources and identifying opportunities for improvement. For example, auditability and traceability improved by blockchain can help streamline insurance claim processes and make them easier to manage. Blockchain can also significantly reduce administration costs and potentially eliminate some intermediaries that were previously needed for data integration. Aggregated health care data can help health care organizations reconfigure their procedures and innovate medical services for patients. With enhanced traceability and transparency supported by blockchain, organizations can learn how to optimize the health care supply chain.

### Interrelationships Among Perspectives

The BSC does not explicitly consider the interactions and trade-offs between perspectives. In dynamic environments, correctly identifying and addressing trade-offs between perspectives can help organizations accurately evaluate the target system and develop effective incentives to improve overall organizational performance. Focusing on the financial perspective alone may motivate organizations to reduce nonfinancial investments that could produce long-term benefits. In particular, if a nonfinancial perspective has no contemporaneously congruent relationship with financial perspective, managers may reduce investments that improve performance in other areas for short-term benefits.

Our approach suggests that in addition to evaluating value-based care with respect to each perspective, health care organizations need to examine the interrelationship among the five perspectives. For example, efforts to improve the efficiency of internal processes (eg, improving quality process within a unit) with blockchain applications can help health care organizations enhance their learning capabilities (eg, creating quality management processes at the organizational level).

While developing relevant key metrics for each perspective (see Multimedia Appendix 3) is crucial for the effective use of the BSC, it is also important to carefully examine the relationships among the perspectives to understand how focus on one affects performance in others in both the short and long term (see Multimedia Appendix 4 for some of the tradeoffs that merit consideration). The relationship is dependent on case characteristics and is therefore not conclusive. For example, as health care organizations learn how to better use blockchain applications, they can use this knowledge to improve their internal processes. Efficient and effective processes can lead to improved service quality, thereby increasing customer satisfaction and revenue in the long term. In turn, organizations can invest more resources in identifying opportunities to learn and develop blockchain applications across the various units. Similarly, an existing health care system may provide a moderate level of data protection that can be achieved with minimal investment, moderate levels of customer satisfaction, and minimal changes to internal processes and learning capabilities. When providing more secure protection of patients’ medical data becomes a top priority for compliance with external and regulatory requirements, organizations may consider adopting a blockchain solution. From the financial perspective, adopting blockchain applications may have a negative impact on organizations as it increases costs in the short term. In addition, blockchain adoption may decrease customer satisfaction in the short term until customers become familiar with the new systems and realize value through capabilities such as ease of access and control. These short-term negative impacts from the customer and on financial perspectives may delay the adoption of improvements to internal processes. In the long term, however, improvements to internal processes that are facilitated by the technology may positively affect customer satisfaction. In addition, process improvements can facilitate learning capabilities, which, in turn, positively affect internal processes and organizational finances in the long term.

### Case Study: Analysis of the Proposed Extended Balanced Scorecard with a Blockchain Implementation in Health Care

#### Outline

What follows is a case study applying the BSC framework to the implementation of a blockchain in health care. PharmaChain Inc is a business unit that manages aviation and trucking transportation within the supply chain journey for pharmaceuticals. PharmaChain Inc prides itself on maintaining pharmaceutical supply chain industry certification to handle high-value, temperature-sensitive cargo. The highest impact of blockchain implementation is providing greater visibility and transparency, thereby ensuring the safe transportation of life-saving pharmaceuticals. Business leaders suggest that this blockchain application use case, managing aviation and trucking of pharmaceutical products from manufacturers to health care providers, serves as an example of PharmaChain Inc’s commitment to pursuing high impact innovation.

**Figure 2:**
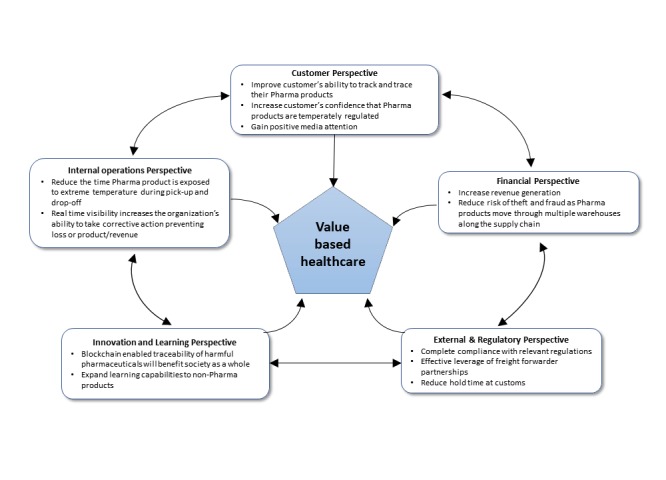
Case study: Application of developed framework in the pharmaceutical supply chain.

While stakeholders often have varying perspectives and goals, this use case illustrates significant benefits for two important stakeholder groups, namely, customers and providers. The varying stakeholder goals within supply chains results in operational complexity when the process is desynchronized. Blockchain technology helps standardize stakeholder interactions, contributing mutual benefit to the provider and the customer. Standardization of interactions, in turn, reduces human intervention and results in accrual of added business value to all stakeholders. See Figure 2 for a summary of the case study.

#### Customer Perspective

Customers are at the center of all decisions at PharmaChain Inc. PharmaChain Inc is committed to customer service and innovation, and these two values guide its decision to strengthen its pharmaceutical transportation services. The blockchain solution enables customers to track and trace their temperature-regulated pharmaceutical products, thereby increasing consumer confidence. As an additional benefit, the organization receives positive media attention regarding its commitment to safely transporting pharmaceutical products, which positively strengthens the company’s relationship with its customer base. Thus, the customer gains real-time access via mobile device or desktop computer to trustworthy information via the blockchain, and the need to contact customer service, which can be time consuming for the customer and costly for the company, is removed.

#### Internal Perspective

PharmaChain Inc explores various parts of the internal and external process to solve customer challenges. Blockchain aids in the reduction of lags in the internal processes between temperature measurement and timely corrective actions. Those lags may have otherwise resulted in increased liability, loss of product efficacy, and product destruction. This implementation facilitates the monitoring of the pharmaceutical products’ exposure to undesirable conditions (such as temperature extremes and delays in transit).

#### External Perspective

Conversely, blockchain delivers external process improvements leveraged to resolve legal and compliance issues more rapidly, ultimately allowing lifesaving medicine to reach patients more quickly by eliminating customary hold times in customs. A blockchain initiative was selected to improve visibility and facilitate trust among stakeholders (eg, manufacturers, distributors, transporters, government agencies, and pharmacies). If the freight forwarders do not produce and submit customs approval forms in a timely fashion, the pharmaceutical products cannot be released, with the duration of the hold potentially affecting the quality of the product and negatively affecting the customer experience. A trusted blockchain minimizes the standard 4- to 8-hour hold duration necessary to verify the validity of the customs approval submitted by the freight forwarder, and improved compliance also helps increase trust among external partners.

#### Learning and Growth (Innovation) Perspective

Blockchain applications support PharmaChain Inc in improving its learning capabilities by enabling it to analyze its business processes and optimize them. The learning capabilities can be extended beyond pharmaceutical products, resulting in organizational efficiencies. The growth opportunity within blockchain applications is enabling traceability along the supply chain journey. Traceability helps reduce fraud in the pharmaceutical supply chain, which is a major societal benefit. Encouraged by the success of the initiative, the organization is deploying blockchain across multiple business products, especially for high value activities and products like pet transportation and food items.

#### Financial Perspective

For PharmaChain Inc, pharmaceuticals represent one of its highest grossing revenue centers among all its shipping products. With a supply chain industry ripe for innovation, PharmaChain Inc accepts that a financial investment must be made to realize the key benefits of blockchain technology. Blockchain technology reduces the risk of theft and fraud as pharmaceutical products move through multiple warehouses along the supply chain, thus justifying the financial investment. The blockchain solution implemented by PharmaChain Inc positively impacts customer service and internal and external processes, increasing reliability and thereby reducing long-term costs. The risk of theft is minimized due to the automation of security controls, facilitated by the blockchain implementation. In addition, the cost of physical tracking of shipments is also minimized. The organization anticipates a lift of 10% in pharmaceutical sales over an 18-month period due to the initiative.

#### Tradeoffs Between the Perspectives

Transparency is one of the key characteristics of blockchain that helps to facilitate value within health care. Transparency helps ensure the authenticity of the pharmaceutical products while providing a lone source of the truth for the pharmacy supply chain network. However, transparency comes with tradeoffs between the value-based perspectives. For example, transparency replaces the concept of *need to know* that previously existed between the internal operational perspective and the customer perspective. Prior to the adoption of the blockchain solution, process improvements that were necessary to address internal operational failure were implemented only when the benefits outweighed the costs. With the introduction of blockchain, increased transparency may increase the exposure of failures in internal operations to the entire supply chain network, which, in turn, may reduce confidence in PharmaChain Inc. Therefore, any deficiencies identified in internal processes will be addressed more rapidly. While this increases the cost of the pharmacy product in the short term, it is likely to improve performance in the long term. Since blockchain in pharmaceuticals is transformational in providing trusted information, positive media attention that results from being an innovator in the industry provides additional opportunities for expanding the customer base.

Thus, PharmaChain Inc needs to continuously balance competing demands to improve internal operations and to innovate. Blockchain innovations require financial and human capital investments, which compete with the demands to improve existing internal systems. Thus, at least in the short term, increased quality of services provided to the customer (for example, via the ability to track and trace pharmaceutical products) may negatively affect the financial metrics. However, the benefits are expected to significantly increase financial performance in the long term as the blockchain technology enables PharmaChain Inc to offer superior services in comparison to its competition, thus providing PharmaChain Inc the opportunity to strengthen its competitive position in the industry.

## Discussion

Thus, we provide a comprehensive framework that can be used to evaluate blockchain implementation in the value-based health care context, and our study contributes to research streams on blockchain technology, the balanced scorecard framework, and value-based care.

First, our framework can help decision makers in health care organizations evaluate the feasibility and utility of various blockchain proposals that seek to address the health IT chasm reported in prior research [1]. We examined the health IT chasm from three stakeholder perspectives to identify how blockchain-based solutions can resolve these issues based on existing use cases (Multimedia Appendix 1). However, because this disruptive technology is still in its infancy, having a holistic view of the value of blockchain applications is critical to making informed strategic investment decisions [55,81]. Our framework will aid health care organizations in holistically considering the implications of blockchain technology from five critical perspectives. While prior literature has identified three groups of stakeholders central to the delivery of value-based care [1], our study additionally highlights the critical role of external stakeholders and regulations.

In addition, our study extends the BSC framework by emphasizing the importance of the external perspective within the health care domain. The health care domain is a dynamic environment marked by changing regulations as well as competitive forces that are charting the course of the industry more rapidly than ever before. Regulatory compliance and value-based provision of services and products are two salient considerations in the health care industry. While value can be created through external partnerships, interoperability among IT systems and regulatory compliance are two areas of concern that constrain such partnerships. Blockchain’s inherent characteristics, such as transparency, immutability, and traceability, facilitate interoperability and enable health care organizations to both cocreate value with their external stakeholders and comply with regulations. Considering the influence of the external environment on a health care organization’s existence, our framework enables the examination of the external perspective when evaluating the performance of blockchain-based HIT solutions.

Third, with their emphasis on value-based care, health care organizations need to develop integrated health care IT infrastructure that can improve services and reduce medical errors. Blockchain, with its inherent trust- and security-promoting qualities, has the potential to significantly affect various areas of value provision for patients in health care. While many performance evaluation solutions exist, our study demonstrates the unique aspects of BSC in evaluating IT initiatives for enabling value-based care. The BSC framework enables the consideration of both financial and nonfinancial dimensions of IT initiatives in the short term as well as the long term. When compared with other performance evaluation solutions (such as Zachman’s framework, the HCI framework, or the technology-centric framework), our extended BSC framework facilitates consideration of the external perspective. It also defines and assesses performance against operational metrics for each of the five critical perspectives. In addition, our approach highlights the importance of the interrelationships among the perspectives, thus offering another critical extension of the BSC approach. The BSC, however, is limited in its ability to build intuitive and interactive systems like those that HCI and other frameworks provide. Thus, we recommend combining the BSC approach with other appropriate framework(s) to meet an organization’s unique needs.

Finally, our case study illustrates how the proposed framework can be utilized to evaluate a health care blockchain application in the for-profit sector. Our approach can also be extended to not-for-profit organizations, which prioritize social goals over financial goals. In such organizations, the financial perspective can be modified to focus on financial sustainability by establishing metrics such as cost reduction, revenue growth, and cost of stakeholder engagement. Similarly, the customer perspective may be widened to include additional stakeholders, such as donors, funding sources, community, volunteers, and employees, that are critical to such organizations [82].

## Appendix

Multimedia Appendix 1 How blockchain can empower value-based care.

Multimedia Appendix 2 Assessment of performance evaluation frameworks.

Multimedia Appendix 3 Metrics (KPIs) per perspectives.

Multimedia Appendix 4 Relationship matrix among perspectives.

## Abbreviations

BMPLS: Blockchain-Based Multi-level Privacy-Preserving Location Sharing Scheme
BSC: Balanced Scorecard
EHR: electronic health record
EFQMEM: European Foundation Quality Management Excellence Model
GDPR: European General Data Protection Regulation
HCI: human-computer interaction
HIT: Health Information Technology
IS: Information Systems
IT: Information Technology
ONC: Office of the National Coordinator for Health Information Technology
ROI: return on investment
TQM: total quality management
