# Supplementary material for: Benefits of Blockchain Initiatives for Value-Based Care: Proposed Framework
Source: J Med Internet Res. 2019 Sep 27;21(9):e13595. doi: 10.2196/13595 (PMC6789420; doi:10.2196/13595)
Supplement: Multimedia Appendix 1 [file jmir_v21i9e13595_app1.pdf]

	Health IT Gaps	Goal	What Blockchain Can Do	Examples
**Patient Perspective**	Patients are unable to access past medical records electronically [20].	Improve patient access to clinical data [1].	Blockchain can serve as the core infrastructure of electronic health records.<br>• Blockchain can eliminate the need for a centralized provider to own patients' health data via decentralization.<br>• Patients may access their health data, which may be stored off-chain or on-chain and linked to the patients' public keys.<br>• Smart contracts can help patients develop access rules for their data, such as allowing a dentist to access dental records for a fixed period [21].	Medical Chain, the first EHR based on blockchain, is able to give patients ownership of patient health information rather than doctors or medical facilities [22].<br><br>MedRec, another blockchain-enabled platform, aims to give patients agency over their own data. Through fine-grained access permissions built on blockchain, patients can determine who can access their data [23].
	Most healthcare providers don't provide functionalities for patients to submit patient-generated data online [1].	Improve patient capability to submit data and access data via mobile health and related technologies [1].	• Blockchain has been proposed as an approach for storing, sharing, and retrieving remotely collected biomedical data [21][24][25]. Public-key infrastructure (PKI) ensures trusted identification of patients by providers and institutions [21].	An mHealth system for cognitive behavioral therapy for insomnia was developed using a smartphone app together with a blockchain storage platform; the tamper resistance of the data was validated [25].<br><br>SMEAD, a mobile-enabled device, deploys blockchain to monitor diabetes patients [26].
**Provider Perspective**	Lack of standardized application interfaces constrains clinician access to external data and knowledge and to advanced analytics that can enable the integration of personalized assistance into the clinical workflow [27].	Enable interoperability with APIs [1].	• With institution-specific logins via patient portals, patients can connect to different institutional interfaces and offer their blockchain public keys as well as their permission to securely transmit data or meta data to the blockchain. Patient health data can therefore be aggregated and accessed across multiple institutions by doctors using blockchain technology [21][28][29].	A blockchain-based framework [30] that utilizes smart contracts is being built on the Ethereum platform to achieve interoperability of electronic health records and to ensure data security, privacy, and access control.
	Before a physician officially joins an office, network, or hospital, these organizations must confirm the physician's credentials, such as education and licensing. It takes more than 120 days for physician data, payment and contract information to be manually checked, reconciled, and audited [31].	Simplify the credentialing and documentation process.	• With immutability and smart contracts, blockchain can streamline enrollments, payments, and contracts.<br>• Blockchain-enabled systems would permit data related to provider and payer credentialing to be shared and updated in nearly real time. Processes that used to take weeks or months can be shortened to a few days.<br>• Credentialing information can be immutably stored on blockchain. Blockchain transactions can be coded into smart contracts, thereby reducing the time it takes to make payments, audit them, fulfill contract terms, and detect potential fraud [8].	Funk et al. [32] presented a blockchain-based application in health professional education (HPE) that offers improved tracking of content and the content's creator as well as certification and credentialing of healthcare professionals without intermediaries [32].

	Healthcare providers are unsure about the authenticity of pharmacy products [33].	Increase transparency of the supply chain.	Via transparency, traceability, and immutability, blockchain enables the tracking of products through the supply chain using track-and-trace and serialization technology [33].<br>• Recording every transaction relating to the prescription of drugs on blockchain so that all stakeholders, such as manufacturers, distributors, care providers, patients, pharmacists, and patients, are connected.<br>• Any malicious modification of the prescription by any stakeholder can be detected [1].	Hyperledger Fabric launched the Counterfeit Medicines Project to combat the challenges associated with counterfeit drugs [34]. Another blockchain-based application for pharmaceutical supply chain management, documented by Bocek et al., uses blockchain to achieve data immutability and public accessibility of the temperature records of pharmaceutical products throughout the supply chain process to verify compliance with requirements [35].
**Researcher and innovator perspective**	Only a small percentage of patients receive clinical trial information from their primary physician [1]. It is rare for providers to participate in biobanks [31].	More readily engage patients in clinical research [1].	• Blockchain's inherent anonymization of data makes it easier for patients to grant permission for their data to be utilized for clinical trials.<br>• The transparency and public nature of blockchain make it easier to replicate blockchain-based clinical research.	To solve this problem, a blockchain protocol proof-of-concept of content traceability in clinical trials has been implemented [36].<br><br>Nugent et al. [37] developed an approach to improve data transparency in clinical trials using a system built on the Ethereum blockchain platform.<br><br>Benchofi and Ravoud conducted a proof-of-concept experimental study to implement a blockchain system to collect participants' consent. They timestamped each patient consent on the blockchain and asked for renewal for protocol revision. The consent and protocol data are hashed into a cryptographic form that provides a secure and robust proof of existence of the entire consent collection process [38].
	The health IT environment is immature and has few safeguards for safety and effectiveness and limited integration of apps into clinical care or research.	Increase the security, effectiveness, and integration of applications.	• Some blockchain cryptographic schemes are proposed to strengthen the security and validity of stored health data.<br>• For example, an attribute-based signature scheme with multiple authorities is proposed. It enables patients to endorse a message to be appended to the blockchain based on attributes of the message, without disclosing any sensitive information.<br>• This protocol has been demonstrated to resist collusion attack and is computationally secure [39].	Zhang and Lin proposed a blockchain-based secure and privacy-preserving EMR system utilizing private blockchain and consortium blockchain to store the actual EMR and the pointers to the EMR, respectively [40].<br><br>Healthcare Data Gateway (HDG) allows patients to control and share their data without violating patients' privacy [41].<br><br>Multi-signature blockchain contracts are also utilized to achieve access control and data privacy [42].
