# Supplementary material for: Benefits of Blockchain Initiatives for Value-Based Care: Proposed Framework
Source: J Med Internet Res. 2019 Sep 27;21(9):e13595. doi: 10.2196/13595 (PMC6789420; doi:10.2196/13595)
Supplement: Multimedia Appendix 2 [file jmir_v21i9e13595_app2.pdf]

<table>
<tr><th rowspan="2">Goals of Blockchain-based HIT Solutions</th><th rowspan="2">Functional Area (that could be impacted)</th><th colspan="8">Framework Assessment</th></tr>
<tr><th>Zachman Framework</th><th>HCI</th><th>Technology – centric Framework</th><th>TQM</th><th>EFQMEM</th><th>Performance Pyramid</th><th>Performance Prism</th><th>Balance Score Card</th></tr>
<tr><td>Improve patient access to data generated by mobile Health and related technologies</td><td>• Customer service
• Business IT Systems & Infrastructure
• Regulatory requirements compliance</td>
<td rowspan="6">• Evaluates business-IT alignment in detail.
• Lacks an external perspective
• Lacks a holistic governance framework with an integrative measurement methodology.
• No explicit consideration of inte-relationships between each of the area impacted.</td>
<td rowspan="6">• Supports the development of intuitive and interactive systems.
• Does not include external and financial perspectives.
• No explicit consideration of inte-relationships between each of the area impacted.</td>
<td rowspan="6">• Considers organization, technology and process simultaneously rather than in an isolated manner.
• More emphasis on the technology aspect with lesser emphasis on other resources used in creating value.
• Does not consider the internal, external, customer perspectives distinctively, thereby affecting the clarity of the impact of disruptive technology on each perspective.</td>
<td rowspan="6">• Integrates the performance management and control systems
• Focuses more on the internal processes of systems used in organizations, the concept of empowering people [63].
• “TQM does not consider employees’ satisfaction in its search for continuous improvement, but the BSC does.” [63]</td>
<td rowspan="6">• Is used to carry out self-assessment as a benchmark of the organization’s position among competitors.
• Assesses performance against a standard set of activities or generic “best practices”.
• Lacks focus on strategy.</td>
<td rowspan="6">• Builds four levels that link corporate strategy, strategic business unit and operation.
• In the use of strategic map, it is less effective than the BSC [64].
• The success map of the Pyramid Performance is more difficult to understand than the strategy map of BSC [64].</td>
<td rowspan="6">• Focuses on the involved stakeholders through five perspectives: satisfaction, strategies, processes, capabilities, and contributions.
• Neglects issues such as how the performance measures can be realized, and therefore, little consideration is given to the processes of designing the system [65].</td>
<td rowspan="6">• Enables performance measurement of financial and non-financial perspectives of a given strategic initiative.
• Defines and assesses performance against operational metrics for each of the perspective to provide a holistic overview.
• Lags on the technical aspects to be considered for building intuitive and interactive systems.</td></tr>
<tr><td>Engage patients in clinical research.</td><td>• Customer engagement
• Internal operations
• External partnerships
• Regulatory requirements compliance</td></tr>
<tr><td>Enable interoperability within an API context</td><td>• Business IT Systems & Infrastructure
• External partnerships
• Regulatory requirements compliance</td></tr>
<tr><td>Simplify the documentation and quality measure process</td><td>• Internal operations
• Regulatory requirements compliance</td></tr>
<tr><td>Increase transparency of the supply chain</td><td>• Internal operations
• External partnership
• Regulatory requirements compliance</td></tr>
<tr><td>Simplify the credentialing and documentation process</td><td>• Internal operations
• Regulatory requirements compliance</td></tr>
</table>
