# Supplementary material for: Benefits of Blockchain Initiatives for Value-Based Care: Proposed Framework
Source: J Med Internet Res. 2019 Sep 27;21(9):e13595. doi: 10.2196/13595 (PMC6789420; doi:10.2196/13595)
Supplement: Multimedia Appendix 3 [file jmir_v21i9e13595_app3.pdf]

Perspectives	Metric / KPI
Financial	Return on Investment (ROI)<br>Rate of revenue growth<br>Rate of cost reduction
Customer	Level of customer satisfaction<br>Level of customers' data access<br>Easiness level of customers' data access
Internal Operation	Visibility level for corrective actions<br>On-budget delivery<br>Increased accuracy of diagnosis<br>Increased interoperability of healthcare IS
Innovation and Learning	Traceability level of pharmaceuticals<br>Training days / person
External & Regulatory	Compliance level with HIPPA<br>Administration cost to comply with HIPPA<br>Compliance costs for GDPR<br>Rate of data breach
