# Supplementary material for: Benefits of Blockchain Initiatives for Value-Based Care: Proposed Framework
Source: J Med Internet Res. 2019 Sep 27;21(9):e13595. doi: 10.2196/13595 (PMC6789420; doi:10.2196/13595)
Supplement: Multimedia Appendix 4 [file jmir_v21i9e13595_app4.pdf]

Perspectives	Finance	Customer	IO	IL	ER
Finance (F)		F->C<br>ST (-) LT (+)	F->IO<br>ST (-) LT (+)	F->IL<br>ST (-) LT (+)	F->ER<br>ST (-) LT (+)
Customer (C)	C->F<br>ST (-) LT (+)		C->IO<br>ST (+) LT (+)	C->IL<br>ST (-) LT (+)	C->ER<br>ST (-) LT (+)
Internal Operation (IO)	IS->F<br>ST (-) LT (+)	IO->C<br>ST (+) LT (+)		IO->IL<br>ST (+) LT (+)	IO->ER<br>ST (-) LT (+)
Innovation & Learning (IL)	IL->F<br>ST (-) LT (+)	IL->C<br>ST (-) LT (+)	IL->IO<br>ST (+) LT (+)		IL->ER<br>ST (-) LT (+)
External & Regulatory (ER)	ER->F<br>ST (-) LT (+)	ER->C<br>ST (-) LT (+)	IL->ER<br>ST (-) LT (+)	ER->IL<br>ST (-) LT (+)	

ST: short term; LT: long term
++: positive relation for both short and long term.
-+: negative relation for a short term. positive relation for a long term.
